# Supplementary material for: Transcript-dependent effects of the CALCA gene on the progression of post-traumatic osteoarthritis in mice
Source: Commun Biol. 2024 Feb 23;7:223. doi: 10.1038/s42003-024-05889-0 (PMC10891124; doi:10.1038/s42003-024-05889-0)
Supplement: Supplementary file 2 — Supplementary Figs. [file 42003_2024_5889_MOESM2_ESM.pdf]

Supplementary Figure 1

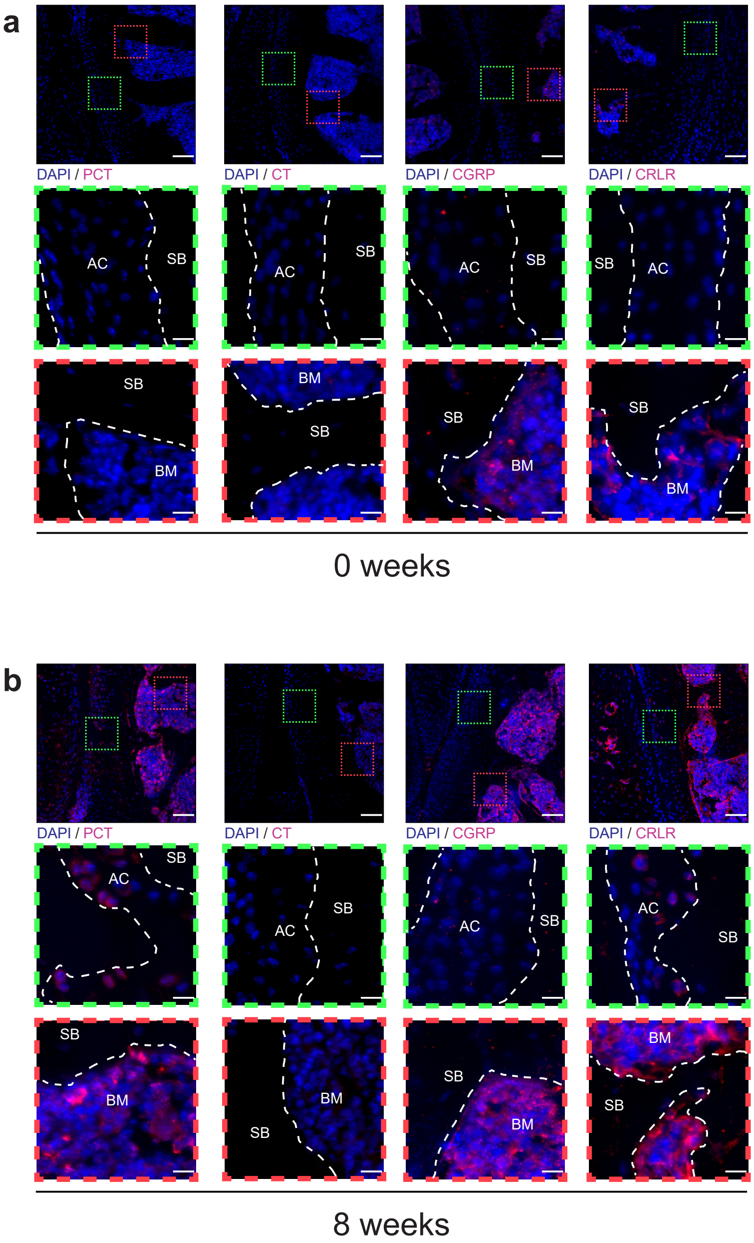

Supplementary Figure 1. Protein expression of *CALCA*-encoded peptides and CRLR in ptOA knees of WT mice. a Representative images of immunofluorescent stainings specific for PCT, CT, αCGRP, and CRLR in the proximal tibia of the unoperated (0 week) and **b** diseased knees 8 weeks after the induction (8 weeks). Site-matched images with higher magnification are presented in red and green boxes. Subchondral bone (SB), articular cartilage (AC) and subchondral bone marrow (BM) are indicated (scale bar =1st row 100 μm; 2nd and 3rd rows 25 μm).

Supplementary Figure 2

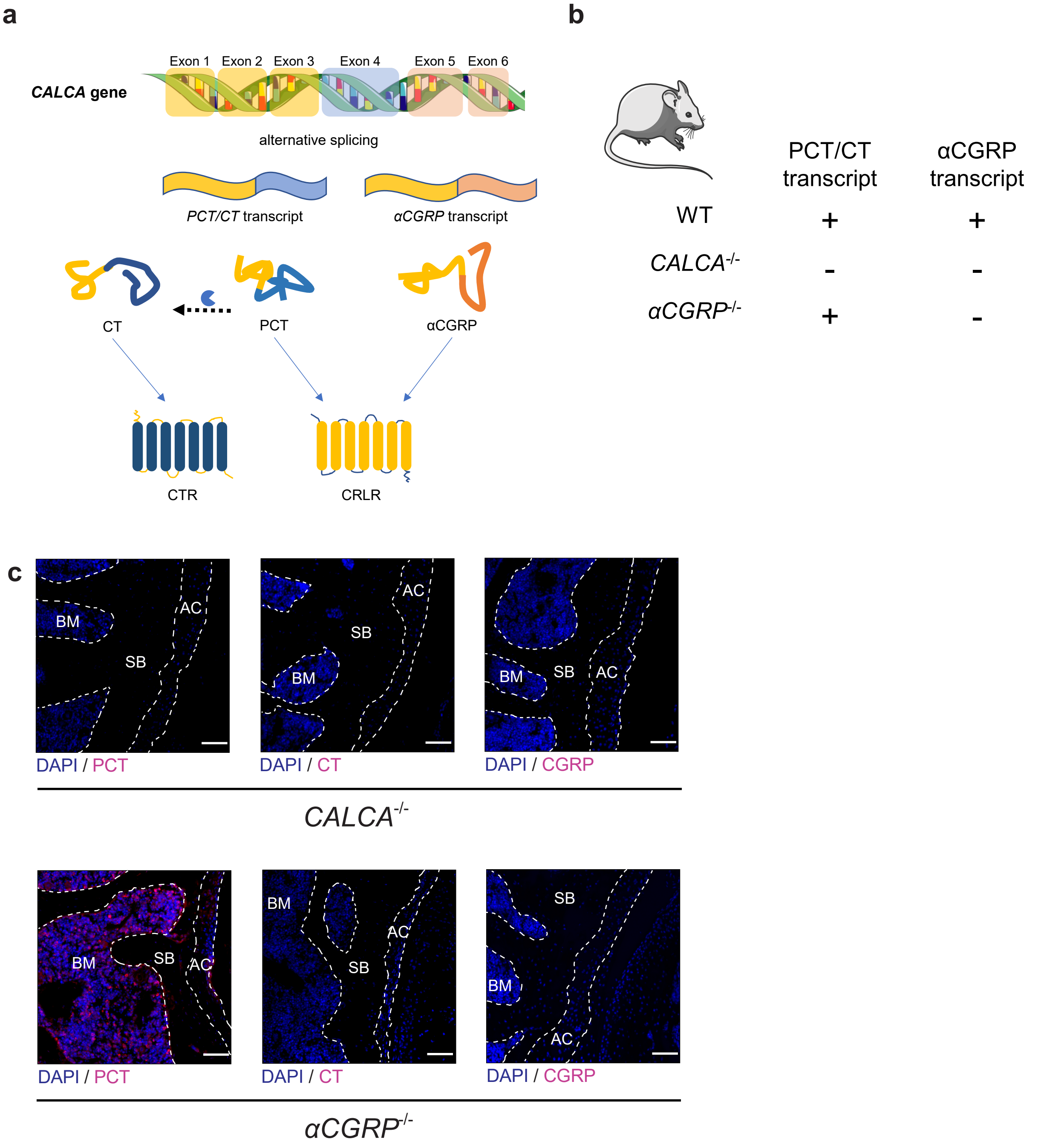

Supplementary Figure 2. Schematic representation of *CALCA* gene alternative splicing and confirmation of transcript deficiencies in the employed mouse strains. a Alternative splicing of the *CALCA* gene results in the transcription of both *PCT/CT* and *αCGRP* mRNA. In thyroid C cells, PCT is usually cleaved at its N- and C-terminal ends to yield CT. In pathophysiologic settings, other cells incapable of further proteolytic processing overexpress *PCT/CT*, resulting in the local release of PCT. Whereas CT binds to the CTR, PCT and αCGRP mediate their biological actions through the CRLR. **b** Summary of mouse strains that were employed in the present manuscript with respective deficiencies. For **a** and **b**, parts of the figures were generated using Servier Medical Art, provided by Servier, licensed under a Creative Commons Attribution 3.0 unported license. **c** Representative overview images of immunofluorescent stainings specific for PCT, CT and αCGRP of the diseased knees in *CALCA*$^{-/-}$ and *αCGRP*$^{-/-}$ mice 4 weeks after the ACLT. Subchondral bone (SB), articular cartilage (AC) and subchondral bone marrow (BM) are indicated (scale bar = 100 µm). n = 6 biologically independent animals were performed.

Supplementary Figure 3

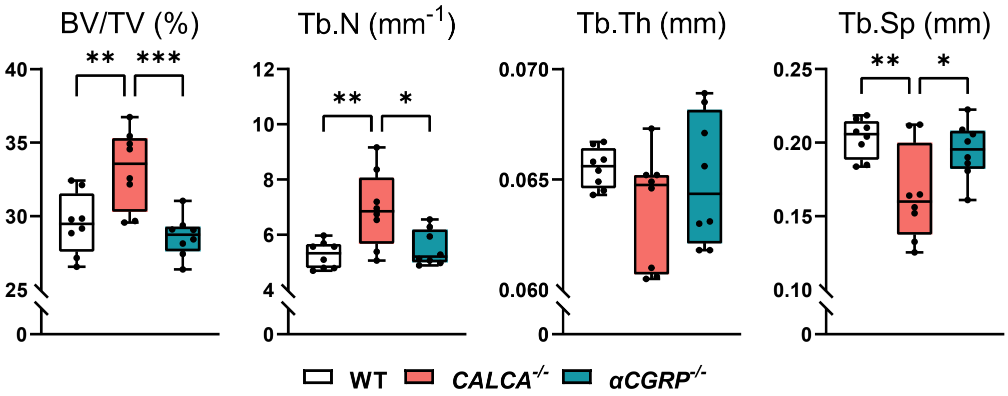

Supplementary Figure 3. Naive *CALCA*- and *αCGRP*- deficient mice exhibit distinct subchondral trabecular bone architecture. Static histomorphometric evaluation of bone volume fraction (BV/TV), trabecular numbers (Tb.N), trabecular thickness (Tb.Th), and trabecular separation (Tb.Sp) of the subchondral trabecular bone in mice of indicated genotypes at the age of 12-14 weeks. n = 8 biologically independent animals as indicated per group and time point. Ordinary one-way ANOVA was used to determine statistical differences. Box plots represent median with minimum and maximum whiskers. *P < 0.05, **P < 0.01, ***P < 0.001.

Supplementary Figure 4

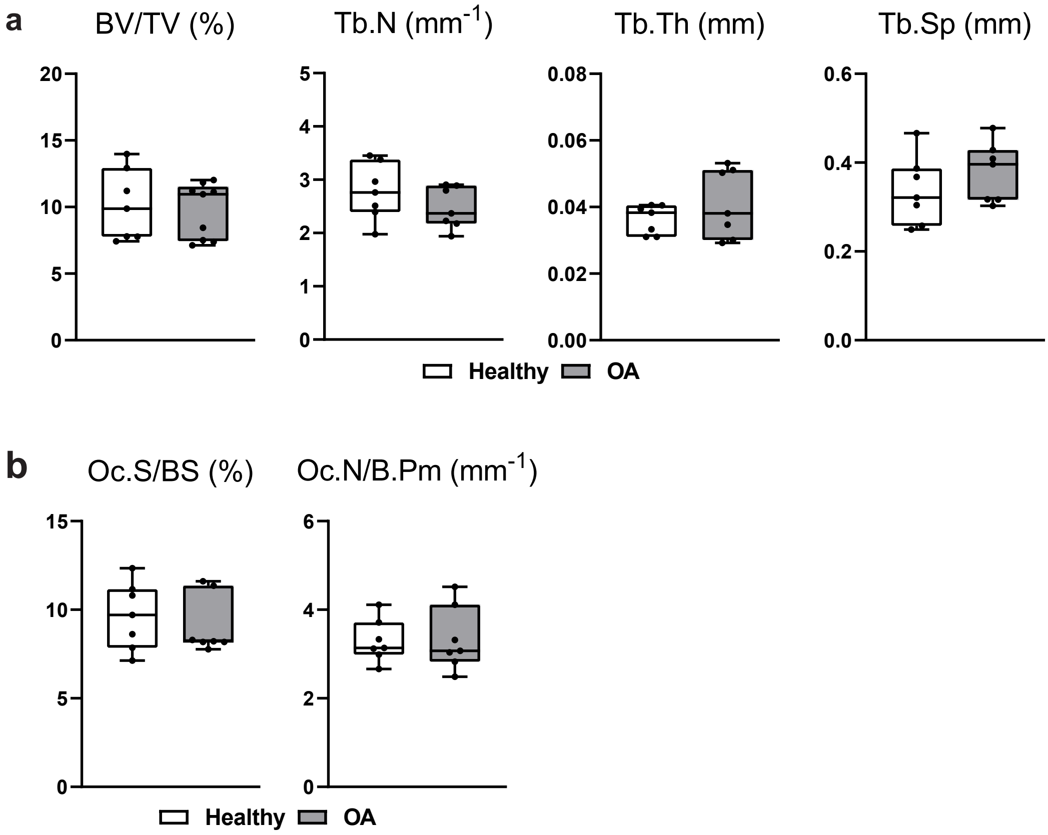

Supplementary Figure 4. ACLT does not affect the metaphyseal trabecular bone structure and bone resorption. a Static histomorphometric analysis of bone volume fraction (BV/TV), trabecular numbers (Tb.N), trabecular thickness (Tb.Th), and trabecular separation (Tb.Sp) of the trabecular bone in the tibial metaphysis of the injured joints and contralateral controls in WT mice 8 weeks after ACLT. **b** Quantification of osteoclast surface per bone surface (Oc.S/BS) and numbers of osteoclasts per bone perimeter (Oc.N/B.Pm) of the metaphyseal trabecular bone at 8 weeks post-operatively. n = 8 biologically independent animals as indicated per group. Unpaired two-tailed students t-test was used to determine statistical differences. Box plots represent median with minimum and maximum whiskers.

Supplementary Figure 5

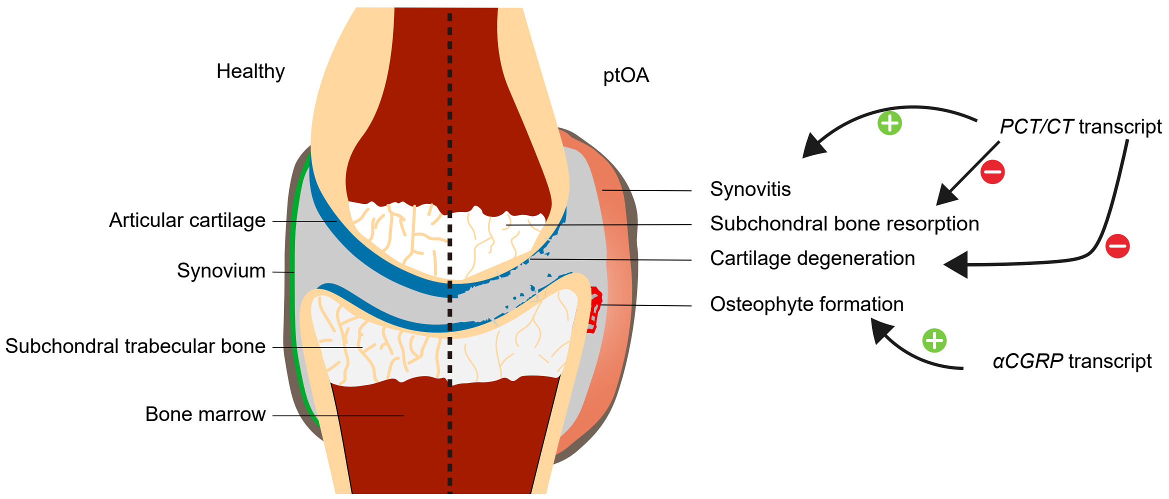

Supplementary Figure 5. Graphic summary of the role of *CALCA* transcripts in ptOA. Cartilage degeneration, abnormal subchondral bone turnover, osteophyte formation and synovitis are key pathophysiological changes of ptOA. During the progression of ptOA, the *PCT/CT* transcript protects from cartilage degeneration and limits subchondral bone resorption, yet aggravates inflammation of the synovial membrane. In contrast, the *αCGRP* transcript functions as a potent driver of osteophyte formation during disease progression in murine ptOA.

Supplementary Figure 6

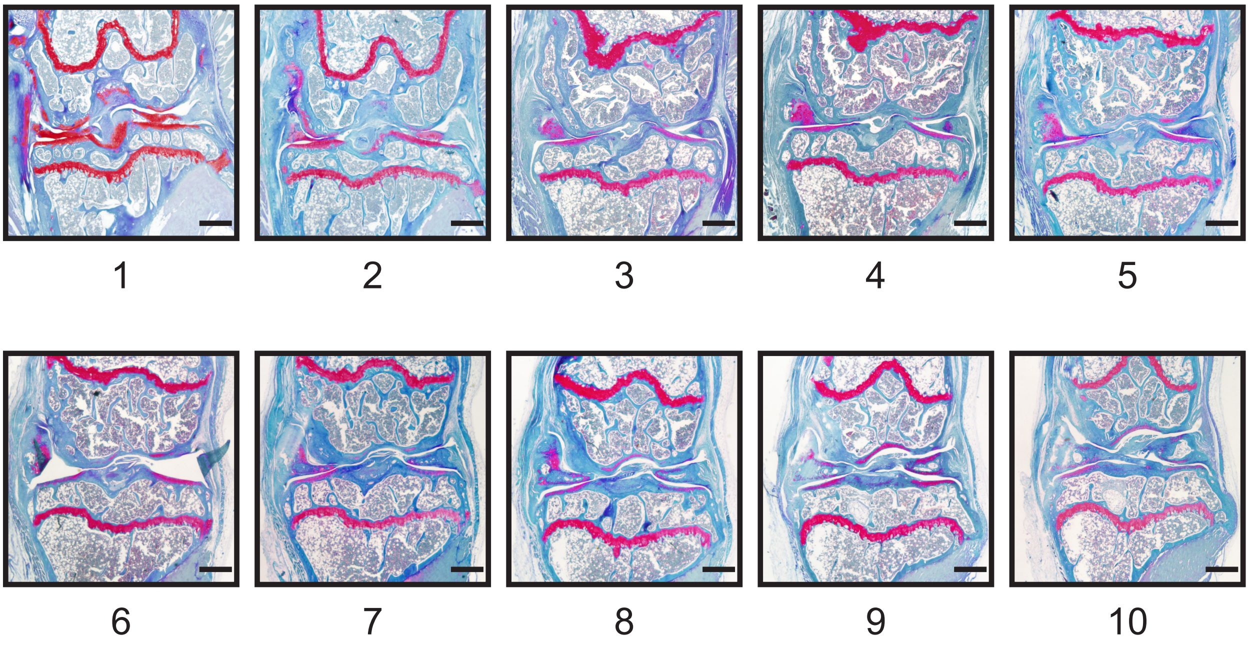

Supplementary Figure 6. Processing of serial coronal sections for OARSI scoring. Representative images of a set of 10 serial sections through the entire diseased knee joint stained with BIC 4 weeks after ACLT (scale bar = 500 μm). The articular cartilage and growth plate stains red, and the bone stains purple/blue. For OARSI scoring, a maximum score was assigned in each section for the four quadrants of the knee joint, including the medial tibial plateau (MTP), medial femoral condyle (MFC), lateral tibial plateau (LTP), and lateral femoral condyle (LFC). OA severity is calculated as the sum of the maximum scores of the total joint, in addition to the individual femoral and tibial quadrant scores, respectively.
