# Supplementary material for: Transcript-dependent effects of the CALCA gene on the progression of post-traumatic osteoarthritis in mice
Source: Commun Biol. 2024 Feb 23;7:223. doi: 10.1038/s42003-024-05889-0 (PMC10891124; doi:10.1038/s42003-024-05889-0)
Supplement: Supplementary file 3 — Description of Additional supplementary Files [file 42003_2024_5889_MOESM3_ESM.docx]

**Description of Additional Supplementary Files**

File name: Supplementary Data

Description: The source data behind the graphs in the paper.
