# Supplementary material for: Transcript-dependent effects of the CALCA gene on the progression of post-traumatic osteoarthritis in mice
Source: Commun Biol. 2024 Feb 23;7:223. doi: 10.1038/s42003-024-05889-0 (PMC10891124; doi:10.1038/s42003-024-05889-0)
Supplement: Supplementary file 5 — Reporting Summary [file 42003_2024_5889_MOESM5_ESM.pdf]

nature portfolio

Corresponding author(s): Johannes Keller

Last updated by author(s): Jan 19, 2024

Reporting Summary

Nature Portfolio wishes to improve the reproducibility of the work that we publish. This form provides structure for consistency and transparency in reporting. For further information on Nature Portfolio policies, see our Editorial Policies and the Editorial Policy Checklist.

Statistics

For all statistical analyses, confirm that the following items are present in the figure legend, table legend, main text, or Methods section.

n/a	Confirmed	
☐	☒	The exact sample size (n) for each experimental group/condition, given as a discrete number and unit of measurement
☐	☒	A statement on whether measurements were taken from distinct samples or whether the same sample was measured repeatedly
☐	☒	The statistical test(s) used AND whether they are one- or two-sided<br>*Only common tests should be described solely by name; describe more complex techniques in the Methods section.*
☒	☐	A description of all covariates tested
☐	☒	A description of any assumptions or corrections, such as tests of normality and adjustment for multiple comparisons
☐	☒	A full description of the statistical parameters including central tendency (e.g. means) or other basic estimates (e.g. regression coefficient) AND variation (e.g. standard deviation) or associated estimates of uncertainty (e.g. confidence intervals)
☐	☒	For null hypothesis testing, the test statistic (e.g. F, t, r) with confidence intervals, effect sizes, degrees of freedom and P value noted<br>*Give P values as exact values whenever suitable.*
☒	☐	For Bayesian analysis, information on the choice of priors and Markov chain Monte Carlo settings
☒	☐	For hierarchical and complex designs, identification of the appropriate level for tests and full reporting of outcomes
☒	☐	Estimates of effect sizes (e.g. Cohen's d, Pearson's r), indicating how they were calculated

Our web collection on statistics for biologists contains articles on many of the points above.

Software and code

Policy information about availability of computer code

Data collection	not applicable.
Data analysis	not applicable.

For manuscripts utilizing custom algorithms or software that are central to the research but not yet described in published literature, software must be made available to editors and reviewers. We strongly encourage code deposition in a community repository (e.g. GitHub). See the Nature Portfolio guidelines for submitting code & software for further information.

Data

Policy information about availability of data

All manuscripts must include a data availability statement. This statement should provide the following information, where applicable:

- Accession codes, unique identifiers, or web links for publicly available datasets
- A description of any restrictions on data availability
- For clinical datasets or third party data, please ensure that the statement adheres to our policy

All relevant data of this project is presented within the figures of this manuscript and its Supplementary Information.

Research involving human participants, their data, or biological material

Policy information about studies with human participants or human data. See also policy information about sex, gender (identity/presentation), and sexual orientation and race, ethnicity and racism.

Reporting on sex and gender	not applicable.
Reporting on race, ethnicity, or other socially relevant groupings	not applicable.
Population characteristics	not applicable.
Recruitment	not applicable.
Ethics oversight	not applicable.

Note that full information on the approval of the study protocol must also be provided in the manuscript.

Field-specific reporting

Please select the one below that is the best fit for your research. If you are not sure, read the appropriate sections before making your selection.

☒ Life sciences ☐ Behavioural & social sciences ☐ Ecological, evolutionary & environmental sciences

For a reference copy of the document with all sections, see nature.com/documents/nr-reporting-summary-flat.pdf

Life sciences study design

All studies must disclose on these points even when the disclosure is negative.

Sample size	The sample size was calculated for the main outcome parameters derived by radiological and histological analysis, yielding a minimum group number of n = 5 mice. In this respect, due to the usual test approaches and laboratory conditions as well as the current lack of prior information (lack of prior probability), realistic error sizes and case numbers were assumed and, with the acceptance of type I and II of 0.05 and 0.2, respectively, group sizes were determined using non-parametric test methods allowing the evidence of effect sizes between 1.5 and 1.8 with a standard deviation of 20%. Due to occasional loss of samples during histologic processing, n = 6-8 per group and time point were used for the main study.
Data exclusions	Outliers are determined via Grubbs' test with a P value > 0.05 and excluded for further analysis.
Replication	All experiments were performed with the involvement of appropriate replicates. Histomorphometric analysis were carried out by 3 experienced researchers independently and all biological assays were conducted with duplicates or triplicates.
Randomization	Animal groups were assigned randomly.
Blinding	Researchers were blinded during sample processing and analyses.

Reporting for specific materials, systems and methods

We require information from authors about some types of materials, experimental systems and methods used in many studies. Here, indicate whether each material, system or method listed is relevant to your study. If you are not sure if a list item applies to your research, read the appropriate section before selecting a response.

Materials & experimental systems

n/a	Involved in the study	
☐	☒	Antibodies
☒	☐	Eukaryotic cell lines
☒	☐	Palaeontology and archaeology
☐	☒	Animals and other organisms
☒	☐	Clinical data
☒	☐	Dual use research of concern
☒	☐	Plants

Methods

n/a	Involved in the study	
☒	☐	ChIP-seq
☒	☐	Flow cytometry
☒	☐	MRI-based neuroimaging

Antibodies

Antibodies used	anti-PCT (LSBio, LS-C296040), anti-CT (Invitrogen, PA5-16464), anti-CGRP (Abcam, ab47027), and anti-CRLR (Bioss Antibodies, bs-1860R-TR)
Validation	The validation of the antibodies are available on the manufacturer's website.

Animals and other research organisms

Policy information about studies involving animals; ARRIVE guidelines recommended for reporting animal research, and Sex and Gender in Research

Laboratory animals	CALCA-/-, αCGRP-/- and WT, all with a C57BL/6J background, were employed in the present study.
Wild animals	Not applicable.
Reporting on sex	Only female mice were employed in the present study. No sex-based analysis was carried out.
Field-collected samples	Mice were housed in a specific pathogen-free animal facility and maintained on standard conditions at a 12 h light/dark cycle and fed ad libitum.
Ethics oversight	All in vivo mouse experiments were performed in accordance with the current recommendations of the "Report of the American Veterinary Medicine Association Panel on Euthanasia" and with approval from the "Behörde für Justiz und Verbraucherschutz-Lebensmittelsicherheit und Veterinärwesen" (N003/2020). We have complied with all relevant ethical regulations for animal use.

Note that full information on the approval of the study protocol must also be provided in the manuscript.
